# Supplementary material for: A content analysis-based approach to explore simulation verification and identify its current challenges
Source: PLoS One. 2020 May 13;15(5):e0232929. doi: 10.1371/journal.pone.0232929 (PMC7219780; doi:10.1371/journal.pone.0232929)
Supplement: S1 Data — (PDF) [file pone.0232929.s001.pdf]

S1 Supplemental. Configuration of the Leximancer tool.

Stage	Category	Variable	Setup
Generate Concept Seeds	Text Processing Settings	Sentence per Block	2 (normal)
		Prose test threshold	1 (default)
		Duplicate Text Sensitivity	Off
		Identify name-like concepts	TRUE
		Break at paragraph	TRUE
		Auto-paragraphing	TRUE
		Merge word variants	FALSE
		Apply folder tags	TRUE
		Apply file tags	FALSE
		Apply dialog tags	FALSE
		File preparation	All Files
		Processed File Storage Format	FastInfoSet Binary XML
	Concept seeds identification	Automatically Identify Concepts	TRUE
		Total Number of Concepts	Automatic
		Percentage of Name-Like Concepts	Automatic
		Concept Specificity	FALSE
		Boilerplate Cutoff	Stronger
Generate Thesaurus	Concept Seeds	Auto concepts	TRUE
		User Defined Concepts	**
	Thesaurus settings	Learn concept thesaurus using source documents	TRUE
		Learn once	FALSE
		Concept generality	12 (default)
		Learn from tags	FALSE
		Learning type	Normal
		Sampling	Automatic
		Phrase separation	3
		Sentiment lens	FALSE
		Number to discovery	Off
		Themed discovery	Concepts in Any
		Only discover name-like concepts	FALSE
