# Supplementary material for: A content analysis-based approach to explore simulation verification and identify its current challenges
Source: PLoS One. 2020 May 13;15(5):e0232929. doi: 10.1371/journal.pone.0232929 (PMC7219780; doi:10.1371/journal.pone.0232929)
Supplement: S2 Data — (PDF) [file pone.0232929.s002.pdf]

S2 Supplemental. Ranked concept list obtained from the Verification Corpus.

The 102 concepts identified from the Verification Corpus as a whole organized by decade. Values shown are concept prominences. Each publication is tagged with decade in which it was published to allow for the calculation of its prominence in each decade (six times total) within the analysis.

The large number of publications comprising the Verification Corpus gives the appearance that prominence values are always very low; however, this is expected since the size of the Corpus directly affects the calculation of prominence.

Concept Name	FOLDER1_1960s	FOLDER1_1970s	FOLDER1_1980s
**activities**	1.3303621	0.5339414	0.7105835
**agent**	0.11461797	0.012048136	0.050187666
**algorithm**	0.30612117	0.8044526	0.5483495
**analysis**	0.47738272	0.8679344	1.0261527
**application**	0.47517502	0.4767792	0.7084508
**approach**	0.2863853	0.5140762	0.7234573
**architecture**	0.061507545	0.025861604	0.3035999
**area**	0.46837336	1.1586245	0.8240736
**average**	0.6488401	1.2807046	0.8178838
**behavior**	0.7748409	0.5194221	1.0859251
**capabilities**	0.15069817	0.43086773	0.5662804
**capacity**	0.8007644	0.85703224	0.6983621
**case**	0.7282358	0.82502735	0.62808096
**code**	0.09867709	0.407985	1.1155429
**complex**	0.5801644	0.748635	0.7788314
**component**	0.45440662	0.4389236	0.7303736
**computer**	5.637897	2.640339	1.8592498
**control**	1.4314673	1.234154	1.0452344
**cost**	1.2576431	1.2711335	0.7836643
**data**	0.58200854	0.9005785	0.8108658
**decision**	0.58954525	1.2577693	1.1038465
**degree**	0.95230186	0.8389481	0.7942543
**design**	0.5490281	0.8148851	1.1180898
**developed**	0.37438333	0.91824883	0.8058555
**development**	0.38515016	0.5351098	0.7025778

**different**	0.3225091	0.5198112	0.71749526
**distributed**	0.31038618	0.32626423	0.60364556
**distribution**	1.7732377	1.5719303	0.82585615
**dynamic**	0.55088043	0.9107053	0.9269776
**dynamics**	0.14972813	0.7974305	0.69932073
**effect**	0.6954923	1.2859349	0.7723395
**effort**	0.4611836	0.8033428	0.98084193
**engineering**	0.4324362	0.4155957	0.6639561
**environment**	0.28969377	0.39282212	0.59849167
**event**	0.6714238	0.7981628	0.9913244
**execution**	0.55361485	0.36503226	0.96964324
**form**	1.3474895	0.91477215	1.1845177
**function**	2.4742365	1.4386451	0.93166345
**future**	0.8530041	0.5760234	0.73054516
**group**	2.4999826	0.60174435	0.71535414
**HLA**	0	0	0
**implementation**	0.1572205	0.55245143	0.7653083
**information**	0.669428	0.6579801	0.7807353
**input**	1.7184485	1.3855355	1.479353
**interface**	0.097976215	0.3295625	0.8840155
**knowledge**	0.1655913	0.3381781	1.0151023
**large**	1.1197902	1.1873094	0.96126264
**level**	0.71986896	0.60535556	0.8418291
**M&s**	0	0	0
**management**	0.657868	0.583121	0.8068512
**method**	1.2285634	0.8741862	0.86993647
**model**	0.40954033	0.9985194	1.1325938
**modeling**	0.13387924	0.55297804	0.8432729
**network**	0.67015696	0.77902704	0.8536465
**object**	0.032112394	0.15527359	0.42950034
**operations**	0.98653483	0.9257138	0.72218996
**order**	0.6830005	0.72857606	0.79247135
**output**	2.1106544	1.331176	1.4189588
**parameters**	0.40879977	1.2676511	0.9942678
**particular**	1.1458409	0.95864964	0.826595
**performance**	0.18150532	0.612914	0.6836704
**policy**	0.47558373	0.83318764	1.1232523
**population**	0.6997844	1.3579983	0.60425687

**power**	0.964939	0.77086896	0.6107291
**problem**	1.5776448	1.1452373	1.1710384
**process**	0.5991664	0.49732226	0.7964327
**product**	0.38106057	0.3547764	0.70208967
**production**	0.921808	0.655914	1.2814586
**program**	2.8298419	2.568767	1.625937
**project**	0.5563588	0.48978657	0.74467844
**quality**	0.3121284	0.8020114	0.58534
**rate**	1.2879666	1.4477943	0.90819645
**requirements**	0.31013116	0.4470805	0.61725837
**research**	0.30081272	0.35069573	0.6368116
**resources**	0.118520565	0.57806784	0.7397609
**result**	0.82663727	1.0084997	0.9011403
**service**	0.29551506	0.74551773	0.62868595
**simulated**	1.245175	1.5270184	1.0031171
**simulation**	0.45829222	0.6840259	0.9465116
**size**	0.8103115	1.2739421	1.0293707
**social**	2.0577173	0.6903133	0.43917742
**software**	0.13339524	0.5404812	0.8814547
**source**	1.0031917	1.0453411	0.68754774
**standard**	0.73121756	0.64783984	0.56550837
**structure**	0.8854967	0.9874521	1.1662599
**study**	0.6871502	0.7736655	0.89474344
**system**	0.61482483	0.9471913	1.1931263
**techniques**	0.80884904	1.2003195	1.2140265
**technology**	0.3963689	0.21211042	0.47907653
**test**	0.4162015	0.77681607	0.8348369
**time**	1.2406639	1.2126411	1.1007806
**tool**	0.32009587	0.40593556	0.7110761
**total**	1.8383026	1.8288242	0.8336835
**training**	0.258729	0.14339948	0.1498042
**type**	1.0798132	1.097989	1.1210757
**unit**	0.92572796	0.9833273	0.876637
**user**	0.7674001	0.8395991	0.9476073
**V&v**	0	0	0
**validation**	0.059732933	0.5588187	0.9296985
**value**	1.6251948	1.1685401	1.0213066
**verification**	0.99332595	0.8121091	1.1387695

**work**	0.93607825	0.407846	0.5864682

FOLDER1_1990s	FOLDER1_2000s	FOLDER1_2010s
0.94045585	1.1000152	0.9871828
0.45894092	1.0492007	1.5925264
1.0098096	0.8779787	1.3039843
1.0111884	0.98389876	1.0310938
0.9806923	1.088337	0.9982841
0.8670603	0.988478	1.2194313
0.8630763	1.2493474	0.9701438
1.0551256	0.97591424	1.0344204
0.74530095	0.8686853	1.3874782
0.8839037	0.9949605	1.1095154
1.0669132	1.2197835	0.785949
0.89380115	0.9080289	1.2896514
0.6784467	1.03026	1.2691401
1.0953	0.905023	1.1185794
0.8150459	0.98143077	1.2273523
0.92138404	1.1786519	0.9013856
1.1730734	0.877048	0.6605052
1.0014	0.9523686	1.033019
0.985976	0.9494695	1.1052883
1.0404075	1.0173944	1.0047361
0.9447016	0.9307512	1.102689
1.0557141	1.030871	0.9768023
1.0864369	0.9348337	1.0374249
1.017802	1.0586525	0.9613793
1.1301532	1.1100639	0.8649587

0.8090023	0.97805667	1.273605
1.1171193	1.1933895	0.7898216
0.99512863	0.8820921	1.1562746
0.88506097	0.9555894	1.1738431
0.62340015	1.0064161	1.3379036
0.8393309	0.98029596	1.167444
1.0811841	1.1756556	0.71420705
0.78640527	1.0867281	1.1449323
0.946959	1.1222631	1.0045263
1.2312117	0.9327593	0.9719764
1.2218293	0.9950114	0.9283373
1.1026697	0.9456748	0.9734335
1.0645183	0.8556462	1.1252956
0.9527973	1.0260268	1.0899374
0.9002981	1.0109848	1.1193731
1.3725234	1.5266478	0.28939345
1.0266596	1.0494578	1.012615
0.94405746	1.1286653	0.92778116
1.0800012	0.84855765	1.0223981
1.3288057	1.087469	0.7494198
0.9147225	1.073848	1.01287
1.0784533	0.9456298	1.0200422
0.87514234	1.0343449	1.1042087
0.6080299	1.2621912	1.1900214
0.9667842	1.0054498	1.0973859
0.8391702	0.9220889	1.2579556
0.93213934	0.9401688	1.1154412
0.8488856	1.0055073	1.1789984
0.8522599	1.0027032	1.1503237
1.8100903	1.0272925	0.6369378
1.0973185	1.0429609	0.93928367
0.8981536	0.9641785	1.1949766
1.1549282	0.8737275	0.94623667
0.870222	0.89993596	1.224526
1.1117969	1.0320567	0.9173668
1.0153793	1.05437	1.0252923
0.50387615	0.9145683	1.4517504
0.47065613	0.821074	1.6800973

0.6218684	0.9751665	1.3930229
1.0975068	0.96453375	0.9283494
0.9787548	1.0748018	0.9964413
0.8195638	1.1387558	1.04289
0.973003	0.93207955	1.0872486
1.2872367	0.9813389	0.53616995
0.9652288	0.9999617	1.1299316
0.7825018	1.10987	1.1001263
0.61541957	0.98204935	1.2590147
1.075384	1.1729269	0.8348767
0.77799547	1.0289589	1.2504767
0.8819392	0.9693823	1.2320926
0.9105714	0.97687835	1.1178607
0.8813439	1.0796088	1.075347
1.081779	0.91874206	1.0181191
1.1212212	1.0011638	0.96582407
1.0505191	0.9004831	1.0890762
0.34091207	0.90398556	1.710595
1.1721355	1.1044121	0.8100879
0.8496635	1.0362241	1.1105546
0.94734484	1.131952	0.96799785
0.8758298	0.9634019	1.1031351
0.79701686	0.922314	1.2979592
1.0285478	0.9631804	1.0046129
1.0488703	0.9487836	0.98345935
1.0687053	1.2066334	0.83763254
1.1818978	1.0541029	0.86429054
1.0155276	0.9382583	1.0376222
1.101221	1.09289	0.9198115
0.7293267	0.8510217	1.3541669
1.098275	1.1968174	0.9136153
1.0383606	0.9881595	0.95595396
1.1148587	0.8998446	1.1034709
1.0428853	1.002201	0.9974172
1.0364705	1.3022768	0.8467007
1.0092229	0.98165184	1.0902508
0.87174064	0.8981084	1.207178
1.1820645	1.0737939	0.75455475

0.8285142	1.011163	1.2387869
