# Supplementary material for: A content analysis-based approach to explore simulation verification and identify its current challenges
Source: PLoS One. 2020 May 13;15(5):e0232929. doi: 10.1371/journal.pone.0232929 (PMC7219780; doi:10.1371/journal.pone.0232929)
Supplement: S3 Data — (PDF) [file pone.0232929.s003.pdf]

S3 Supplemental. Lists of concepts that are prominent alongside verification when we apply content analysis to each decade's publications independently.

Concept prominences and frequencies pertaining to "Verification" in each decade. Content analysis was conducted on each decade using only that decade's publications from the Verification Corpus. Therefore, a total of six content analyses were conducted.

Concepts per decade.

Decade	Number of prominent concepts identified with respect to verification each decade	Number of prominent concepts identified with respect to verification each decade with prominence greater than 0
1960s	54	46
1970s	84	81
1980s	69	69
1990s	77	77
2000s	95	95
2010s	89	89

Notes

* Values of 0 may appear as these concepts have non-0 values with respect to other concepts within their decade.

1960s.

Concept	Prominence with respect to verification	Frequency with respect to verification
verification	66.826	46
solution	8.449	11
results	6.331	9
analog	6.075	15
method	5.503	7
described	5.043	4
case	4.950	4
given	4.455	5
logic	3.819	4
work	3.819	4
order	3.457	3
example	3.398	3
problem	2.688	7

particular	2.673	2
design	2.673	2
available	2.638	3
rate	2.522	2
data	2.404	5
machine	2.228	2
output	2.056	4
model	2.016	7
computer	1.985	9
simulation	1.946	6
value	1.787	5
used	1.694	9
information	1.630	2
dquo	1.604	3
activity	1.530	3
digital	1.412	3
input	1.392	2
social	1.337	1
possible	1.285	1
system	1.280	5
Fortran	1.261	1
individual	1.193	1
level	1.133	1
distribution	1.114	1
facility	1.096	1
function	1.072	3
bone	1.028	1
error	0.997	1
required	0.941	1
time	0.857	4
response	0.857	1
process	0.548	1
program	0.499	1
control	0	0
group	0	0
operation	0	0
behavior	0	0
equipment	0	0
during	0	0
total	0	0
event	0	0

**1970s.**		
**Concept**	**Prominence with respect to verification**	**Frequency with respect to verification**
verification	95.569	153
design	6.072	23
results	5.188	31
program	4.559	28
test	4.518	13
process	4.497	20
software	4.364	10
provide	4.247	10
development	3.995	13
techniques	3.869	10
real	3.240	8
simulation	3.025	51
described	2.911	6
complex	2.626	5
computer	2.564	15
performance	2.553	7
model	2.526	75
approach	2.515	6
used	2.463	44
analysis	2.440	12
changes	2.320	10
research	2.294	3
study	2.285	6
parameters	2.230	7
include	2.223	4
system	2.038	41
required	2.033	8
conditions	2.016	5
work	1.977	3
form	1.850	3
output	1.833	7
effects	1.826	3
random	1.812	4
user	1.798	7
operation	1.707	4
problem	1.688	10
behavior	1.677	3
large	1.641	4
dynamic	1.629	3
flow	1.580	4

operations	1.525	3
data	1.485	16
specific	1.470	3
modeling	1.457	5
response	1.419	3
available	1.377	5
necessary	1.359	3
area	1.309	5
air	1.309	3
information	1.298	5
values	1.285	8
structure	1.161	3
simulated	1.151	3
time	1.124	16
times	1.107	3
input	1.083	4
line	1.068	2
several	1.039	2
important	0.985	2
effect	0.923	2
function	0.913	3
variables	0.890	3
control	0.817	4
period	0.810	2
cost	0.738	2
distribution	0.738	2
level	0.700	2
rate	0.693	2
order	0.678	2
decision	0.661	2
points	0.637	1
aircraft	0.534	1
event	0.522	1
size	0.519	1
particular	0.483	1
network	0.449	1
method	0.440	1
type	0.398	1
possible	0.366	1
total	0.355	1
developed	0.302	1
general	0	0
service	0	0
basic	0	0

**1980s.**		
**Concept**	**Prominence with respect to verification**	**Frequency with respect to verification**
verification	43.482	888
validation	10.795	251
programming	2.980	44
development	2.912	88
computer	2.834	82
application	2.700	48
model	2.634	573
process	2.623	137
design	2.551	90
analysis	2.549	101
program	2.424	65
tools	2.332	37
study	2.183	49
knowledge	2.149	39
developed	2.062	46
software	2.049	54
method	1.920	51
results	1.824	64
complex	1.764	28
based	1.738	41
problem	1.729	81
decision	1.725	38
possible	1.699	41
output	1.687	49
simulation	1.537	229
work	1.495	26
general	1.452	32
base	1.424	30
used	1.401	173
behavior	1.367	36
user	1.364	45
data	1.347	87
support	1.344	25
structure	1.343	31
information	1.335	43
input	1.277	35
language	1.248	39
area	1.185	16
different	1.178	36
system	1.165	181

performance	1.162	28
test	1.154	25
management	1.147	21
flow	1.107	18
approach	1.093	26
components	1.061	18
modeling	1.060	35
available	1.037	27
include	1.016	19
research	0.956	17
level	0.873	27
GPSS	0.847	13
control	0.800	21
processing	0.783	15
event	0.747	18
type	0.692	21
parameters	0.660	13
order	0.654	16
values	0.591	20
network	0.586	12
time	0.571	53
production	0.512	11
variables	0.460	16
machine	0.451	8
function	0.329	6
service	0.321	4
queue	0.287	4
message	0.162	3
rate	0.158	2

**1990s.**		
**Concept**	**Prominence with respect to verification**	**Frequency with respect to verification**
verification	22.997	5015
validation	20.908	3302
M&s	2.933	156
test	2.858	694
model	2.535	2559
development	2.153	533
implementation	2.112	207
results	2.068	417
requirements	2.049	312
behavior	2.018	252
process	1.983	778
application	1.904	223
problem	1.859	315
design	1.778	338
analysis	1.683	366
approach	1.675	265
structure	1.608	174
support	1.581	294
program	1.566	185
software	1.563	294
output	1.553	198
research	1.550	188
data	1.539	743
include	1.530	209
computer	1.509	162
simulation	1.454	1577
distributed	1.418	154
developed	1.414	209
used	1.401	1134
tools	1.252	264
system	1.248	966
modeling	1.241	228
current	1.231	136
described	1.219	121
resources	1.200	101
including	1.198	90
project	1.180	135
level	1.174	195
conditions	1.137	86
provide	1.130	394

degree	1.109	64
training	1.076	111
work	1.067	133
performance	1.046	197
management	1.029	115
components	0.992	111
user	0.954	168
information	0.920	195
input	0.908	110
execution	0.906	95
case	0.904	93
decision	0.884	108
type	0.880	89
HLA	0.847	82
federation	0.846	152
available	0.818	118
environment	0.811	111
operations	0.797	79
target	0.758	55
entity	0.754	68
area	0.753	63
function	0.707	74
control	0.706	99
service	0.694	55
order	0.601	93
aircraft	0.579	33
network	0.561	59
time	0.554	250
values	0.540	86
production	0.538	50
object	0.514	97
RTI	0.510	41
rate	0.477	24
event	0.441	102
class	0.434	28
distribution	0.389	31
message	0.179	22

**2000s.**		
**Concept**	**Prominence with respect to verification**	**Frequency with respect to verification**
verification	26.359	9697
validation	25.043	6811
effort	2.577	348
model	2.253	4404
development	2.243	1030
test	2.237	584
M&s	2.229	446
results	2.221	891
requirements	2.199	653
process	2.186	1837
activities	2.122	362
team	2.015	263
program	1.960	336
design	1.872	567
knowledge	1.795	310
analysis	1.791	727
simulation	1.761	3239
output	1.741	308
implementation	1.737	303
behavior	1.727	431
modeling	1.679	729
developed	1.633	436
software	1.616	494
application	1.600	567
data	1.545	1298
study	1.498	333
support	1.461	461
tools	1.456	552
used	1.452	2387
approach	1.426	447
project	1.415	291
quality	1.404	186
include	1.387	295
problem	1.353	360
degree	1.325	132
research	1.323	347
areas	1.303	148
described	1.303	216
user	1.295	269
engineering	1.273	170

available	1.261	297
system	1.244	1780
experience	1.232	130
dynamics	1.200	186
provide	1.161	526
level	1.154	404
complex	1.141	205
work	1.131	306
structure	1.107	211
agents	1.104	231
current	1.078	227
training	1.075	206
form	1.050	142
input	1.043	168
components	1.020	343
architecture	1.018	156
method	1.009	162
performance	0.983	336
type	0.982	170
product	0.958	345
cost	0.953	154
information	0.947	408
scenario	0.939	154
execution	0.925	163
case	0.909	257
distributed	0.881	183
standard	0.875	146
operations	0.869	147
order	0.858	242
environment	0.853	253
management	0.848	179
area	0.834	115
HLA	0.799	163
capabilities	0.791	138
variables	0.767	111
group	0.711	77
control	0.707	171
technology	0.703	87
value	0.688	215
increase	0.687	85
time	0.669	585
service	0.647	159
entity	0.620	86
resources	0.607	96

event	0.582	119
distribution	0.558	65
received	0.521	48
object	0.515	66
network	0.511	105
function	0.495	74
change	0.478	76
total	0.411	42
capacity	0.381	41
events	0.356	61
rate	0.158	16

**2010s.**		
**Concept**	**Prominence with respect to verification**	**Frequency with respect to verification**
verification	58.124	2991
validation	17.138	1548
M&s	4.163	255
training	4.026	199
requirements	3.831	269
program	3.280	110
development	3.116	361
test	3.010	202
tool	2.486	139
engineering	2.384	109
support	2.381	196
process	2.195	520
software	2.169	162
code	2.123	90
provide	2.079	272
analysis	1.957	261
include	1.922	207
developed	1.921	155
results	1.911	278
application	1.880	125
model	1.879	1385
design	1.860	201
standard	1.845	148
modeling	1.804	258
behavior	1.704	150
simulation	1.691	864
project	1.672	123
data	1.662	437
method	1.642	195
decision	1.603	127
environment	1.583	129
components	1.425	85
used	1.411	702
approach	1.393	170
information	1.359	162
system	1.353	601
structure	1.342	91
distributed	1.306	60
framework	1.300	80
based	1.275	186

complex	1.233	90
output	1.232	80
scenario	1.220	75
social	1.211	59
management	1.190	83
performance	1.190	122
study	1.164	112
level	1.159	134
problem	1.050	93
group	1.043	44
operations	1.042	51
elements	1.016	52
order	0.989	108
input	0.951	61
degree	0.946	29
research	0.945	94
dynamics	0.891	54
control	0.880	73
event	0.861	89
parameters	0.856	62
cost	0.852	48
individual	0.817	46
case	0.802	84
service	0.782	62
different	0.780	122
area	0.777	39
effect	0.773	37
agents	0.743	77
market	0.719	27
energy	0.703	33
type	0.657	36
time	0.649	174
change	0.643	42
resources	0.638	59
variables	0.635	39
value	0.624	84
policy	0.608	39
function	0.568	34
network	0.526	39
production	0.454	27
supply	0.451	21
algorithm	0.447	19
population	0.437	19
patients	0.400	20

rate	0.344	17
water	0.344	8
distribution	0.341	19
power	0.289	13
demand	0.238	12
