# Supplementary material for: A content analysis-based approach to explore simulation verification and identify its current challenges
Source: PLoS One. 2020 May 13;15(5):e0232929. doi: 10.1371/journal.pone.0232929 (PMC7219780; doi:10.1371/journal.pone.0232929)
Supplement: S4 Data — (PDF) [file pone.0232929.s004.pdf]

S4 Supplemental. List of thesaurus terms containing "verification" obtained from conducting separate content analyses on each individual decade within the Verification Corpus.

The complete list of unfiltered thesaurus results for terms associated with *Verification* each decade. Content analysis was conducted on each decade using only that decade's publications from the Verification Corpus (resulting in a total of six content analyses).

Number of related terms per decade.

**Decade**	**Number of Related Terms**
1960s	58
1970s	139
1980s	148
1990s	198
2000s	223
2010s	191

Score Description

* The *score* (column 5) reflects how tightly the *word* (column 4) is connected to the concept or tag (column 2, verification).

* In other words, this is a measure of how strongly the presense of the word (column 46) predicts the appearance of the concept or tag (column 2, verification).

* The score value is derived from the co-occurrence information from the text along with the seeded classifier information.

*Refer to the Leximancer Frequently Asked Questions document for more details (https://www.leximancer.com/faq/display_and_output.html).

Notes

* *iterations* indicates the total number of times that the text corpus was reread and coded with concept definitions before reaching a stable classification.

* *value* indicates that these are the thesaurus terms only pertaining to "verification".

* *kind* column refers to the *value* column (all refer to verification), while *kind2* column refers to the *word* column.

**1960s.**					
**iterations**	**value**	**kind**	**word**	**score**	**kind2**
6	verification	WORD,USER	verification	7.0707555	WORD
6	verification	WORD,USER	accounting	3.992244	WORD
6	verification	WORD,USER	all-digital	3.6686642	WORD
6	verification	WORD,USER	discounting	3.6686642	WORD
6	verification	WORD,USER	advocates	3.12821	WORD
6	verification	WORD,USER	alternating	3.12821	WORD
6	verification	WORD,USER	amazing	3.12821	WORD
6	verification	WORD,USER	assertion	3.12821	WORD
6	verification	WORD,USER	automatically-scaled	3.12821	WORD
6	verification	WORD,USER	circled	3.12821	WORD
6	verification	WORD,USER	coding	3.12821	WORD
6	verification	WORD,USER	computer-asked	3.12821	WORD
6	verification	WORD,USER	concieved	3.12821	WORD
6	verification	WORD,USER	confronting	3.12821	WORD
6	verification	WORD,USER	consolidate	3.12821	WORD
6	verification	WORD,USER	d'etre	3.12821	WORD
6	verification	WORD,USER	declined	3.12821	WORD
6	verification	WORD,USER	deriving	3.12821	WORD
6	verification	WORD,USER	discover	3.12821	WORD
6	verification	WORD,USER	elaboration	3.12821	WORD
6	verification	WORD,USER	emas	3.12821	WORD
6	verification	WORD,USER	enhanced	3.12821	WORD
6	verification	WORD,USER	function&dquo	3.12821	WORD
6	verification	WORD,USER	guides	3.12821	WORD
6	verification	WORD,USER	harder	3.12821	WORD
6	verification	WORD,USER	ignore	3.12821	WORD
6	verification	WORD,USER	impossibility	3.12821	WORD
6	verification	WORD,USER	in-depth	3.12821	WORD
6	verification	WORD,USER	inclusion	3.12821	WORD
6	verification	WORD,USER	influential	3.12821	WORD
6	verification	WORD,USER	investigating	3.12821	WORD
6	verification	WORD,USER	live	3.12821	WORD
6	verification	WORD,USER	measres	3.12821	WORD
6	verification	WORD,USER	met	3.12821	WORD

6	verification	WORD,USER	objection	3.12821	WORD
6	verification	WORD,USER	pencil	3.12821	WORD
6	verification	WORD,USER	ple	3.12821	WORD
6	verification	WORD,USER	precautions	3.12821	WORD
6	verification	WORD,USER	pretend	3.12821	WORD
6	verification	WORD,USER	qecide	3.12821	WORD
6	verification	WORD,USER	quadrature	3.12821	WORD
6	verification	WORD,USER	raison	3.12821	WORD
6	verification	WORD,USER	readability	3.12821	WORD
6	verification	WORD,USER	referenceto-ground	3.12821	WORD
6	verification	WORD,USER	refinement	3.12821	WORD
6	verification	WORD,USER	sam	3.12821	WORD
6	verification	WORD,USER	shooting	3.12821	WORD
6	verification	WORD,USER	spending	3.12821	WORD
6	verification	WORD,USER	stimuli&dquo	3.12821	WORD
6	verification	WORD,USER	strongly	3.12821	WORD
6	verification	WORD,USER	suggest	3.12821	WORD
6	verification	WORD,USER	tilt&dquo	3.12821	WORD
6	verification	WORD,USER	tution	3.12821	WORD
6	verification	WORD,USER	vague	3.12821	WORD
6	verification	WORD,USER	well-being	3.12821	WORD
6	verification	WORD,USER	xy	3.12821	WORD
6	verification	WORD,USER	xz	3.12821	WORD
6	verification	WORD,USER	emphasized	1.9294441	WORD
**1970s.**					

iterations	value	kind	word	score	kind2
6	verification	WORD,USER	verification	8.878786	WORD
6	verification	WORD,USER	assurance	3.9589272	WORD
6	verification	WORD,USER	mathematical-logical	3.7669551	WORD
6	verification	WORD,USER	cussed	3.498189	WORD
6	verification	WORD,USER	intends	3.498189	WORD
6	verification	WORD,USER	verifies	3.498189	WORD
6	verification	WORD,USER	1nlplications	3.0418134	WORD
6	verification	WORD,USER	airplanes	3.0418134	WORD
6	verification	WORD,USER	aleatoric	3.0418134	WORD
6	verification	WORD,USER	and-validation	3.0418134	WORD

6	verification	WORD,USER	aodel	3.0418134	WORD
6	verification	WORD,USER	ascen	3.0418134	WORD
6	verification	WORD,USER	attaches	3.0418134	WORD
6	verification	WORD,USER	auth	3.0418134	WORD
6	verification	WORD,USER	availale	3.0418134	WORD
6	verification	WORD,USER	becaus	3.0418134	WORD
6	verification	WORD,USER	betv1een	3.0418134	WORD
6	verification	WORD,USER	c9st	3.0418134	WORD
6	verification	WORD,USER	capobility	3.0418134	WORD
6	verification	WORD,USER	chaired	3.0418134	WORD
6	verification	WORD,USER	civilian	3.0418134	WORD
6	verification	WORD,USER	cohtinuous-par	3.0418134	WORD
6	verification	WORD,USER	coincides	3.0418134	WORD
6	verification	WORD,USER	compile-time	3.0418134	WORD
6	verification	WORD,USER	concurrency	3.0418134	WORD
6	verification	WORD,USER	connnonly	3.0418134	WORD
6	verification	WORD,USER	consulants	3.0418134	WORD
6	verification	WORD,USER	conveniences	3.0418134	WORD
6	verification	WORD,USER	corrert	3.0418134	WORD
6	verification	WORD,USER	coulq	3.0418134	WORD
6	verification	WORD,USER	developn	3.0418134	WORD
6	verification	WORD,USER	dew	3.0418134	WORD
6	verification	WORD,USER	dfgital	3.0418134	WORD
6	verification	WORD,USER	differentiates	3.0418134	WORD
6	verification	WORD,USER	digitals	3.0418134	WORD
6	verification	WORD,USER	dratnatically	3.0418134	WORD
6	verification	WORD,USER	eaker	3.0418134	WORD
6	verification	WORD,USER	easurement	3.0418134	WORD
6	verification	WORD,USER	echniques	3.0418134	WORD
6	verification	WORD,USER	elers	3.0418134	WORD
6	verification	WORD,USER	equated	3.0418134	WORD
6	verification	WORD,USER	eri	3.0418134	WORD
6	verification	WORD,USER	erlooked	3.0418134	WORD
6	verification	WORD,USER	ewpoint	3.0418134	WORD
6	verification	WORD,USER	exercis	3.0418134	WORD
6	verification	WORD,USER	extensible	3.0418134	WORD
6	verification	WORD,USER	fault-free	3.0418134	WORD
6	verification	WORD,USER	fundation	3.0418134	WORD

6	verification	WORD,USER	genrated	3.0418134	WORD
6	verification	WORD,USER	gratifying	3.0418134	WORD
6	verification	WORD,USER	hiietqrical	3.0418134	WORD
6	verification	WORD,USER	hove	3.0418134	WORD
6	verification	WORD,USER	hurdles	3.0418134	WORD
6	verification	WORD,USER	i'ois	3.0418134	WORD
6	verification	WORD,USER	i'ssile	3.0418134	WORD
6	verification	WORD,USER	iation	3.0418134	WORD
6	verification	WORD,USER	idatlon	3.0418134	WORD
6	verification	WORD,USER	iing	3.0418134	WORD
6	verification	WORD,USER	ilgorithms	3.0418134	WORD
6	verification	WORD,USER	implmentat	3.0418134	WORD
6	verification	WORD,USER	imulator	3.0418134	WORD
6	verification	WORD,USER	in-program	3.0418134	WORD
6	verification	WORD,USER	increses	3.0418134	WORD
6	verification	WORD,USER	incubation	3.0418134	WORD
6	verification	WORD,USER	indepement	3.0418134	WORD
6	verification	WORD,USER	intermittent	3.0418134	WORD
6	verification	WORD,USER	intnded	3.0418134	WORD
6	verification	WORD,USER	invo1ves	3.0418134	WORD
6	verification	WORD,USER	irde	3.0418134	WORD
6	verification	WORD,USER	keypunching	3.0418134	WORD
6	verification	WORD,USER	ldation	3.0418134	WORD
6	verification	WORD,USER	lflying	3.0418134	WORD
6	verification	WORD,USER	logiq	3.0418134	WORD
6	verification	WORD,USER	m9delling	3.0418134	WORD
6	verification	WORD,USER	machine-oriented	3.0418134	WORD
6	verification	WORD,USER	melodies	3.0418134	WORD
6	verification	WORD,USER	mit	3.0418134	WORD
6	verification	WORD,USER	models'of	3.0418134	WORD
6	verification	WORD,USER	mqdel's	3.0418134	WORD
6	verification	WORD,USER	multidisciplinary	3.0418134	WORD
6	verification	WORD,USER	muti-stage	3.0418134	WORD
6	verification	WORD,USER	nessitates	3.0418134	WORD
6	verification	WORD,USER	nicely	3.0418134	WORD
6	verification	WORD,USER	nning	3.0418134	WORD
6	verification	WORD,USER	non-synchronous	3.0418134	WORD
6	verification	WORD,USER	nonrandom	3.0418134	WORD

6	verification	WORD,USER	oras	3.0418134	WORD
6	verification	WORD,USER	othe'r	3.0418134	WORD
6	verification	WORD,USER	outlining	3.0418134	WORD
6	verification	WORD,USER	outweighed	3.0418134	WORD
6	verification	WORD,USER	patchboards	3.0418134	WORD
6	verification	WORD,USER	pel	3.0418134	WORD
6	verification	WORD,USER	perplexing	3.0418134	WORD
6	verification	WORD,USER	pertains	3.0418134	WORD
6	verification	WORD,USER	plies	3.0418134	WORD
6	verification	WORD,USER	post-run	3.0418134	WORD
6	verification	WORD,USER	pr-0pose	3.0418134	WORD
6	verification	WORD,USER	practicably	3.0418134	WORD
6	verification	WORD,USER	precede	3.0418134	WORD
6	verification	WORD,USER	preceded	3.0418134	WORD
6	verification	WORD,USER	pro9ram	3.0418134	WORD
6	verification	WORD,USER	programmer-oriented	3.0418134	WORD
6	verification	WORD,USER	provisioning	3.0418134	WORD
6	verification	WORD,USER	quicker	3.0418134	WORD
6	verification	WORD,USER	r's	3.0418134	WORD
6	verification	WORD,USER	r-e	3.0418134	WORD
6	verification	WORD,USER	re-analysis	3.0418134	WORD
6	verification	WORD,USER	re-verified	3.0418134	WORD
6	verification	WORD,USER	rectitude	3.0418134	WORD
6	verification	WORD,USER	regime	3.0418134	WORD
6	verification	WORD,USER	rification	3.0418134	WORD
6	verification	WORD,USER	routinely	3.0418134	WORD
6	verification	WORD,USER	ruled	3.0418134	WORD
6	verification	WORD,USER	samplirtg	3.0418134	WORD
6	verification	WORD,USER	sarily	3.0418134	WORD
6	verification	WORD,USER	scanty	3.0418134	WORD
6	verification	WORD,USER	self	3.0418134	WORD
6	verification	WORD,USER	simultaneouslv	3.0418134	WORD
6	verification	WORD,USER	slso	3.0418134	WORD
6	verification	WORD,USER	stic	3.0418134	WORD
6	verification	WORD,USER	structur	3.0418134	WORD
6	verification	WORD,USER	stuck-at	3.0418134	WORD
6	verification	WORD,USER	stuck-at-zero	3.0418134	WORD
6	verification	WORD,USER	stuckat-one	3.0418134	WORD

6	verification	WORD,USER	subaccoµnts	3.0418134	WORD
6	verification	WORD,USER	subsidiary	3.0418134	WORD
6	verification	WORD,USER	the'µ's	3.0418134	WORD
6	verification	WORD,USER	three-value	3.0418134	WORD
6	verification	WORD,USER	topical	3.0418134	WORD
6	verification	WORD,USER	truefalse	3.0418134	WORD
6	verification	WORD,USER	tting	3.0418134	WORD
6	verification	WORD,USER	two-point	3.0418134	WORD
6	verification	WORD,USER	valtdity	3.0418134	WORD
6	verification	WORD,USER	witµ	3.0418134	WORD
6	verification	WORD,USER	yerif	3.0418134	WORD
6	verification	WORD,USER	yes-no	3.0418134	WORD
6	verification	WORD,USER	designer's	1.9963038	WORD
6	verification	WORD,USER	fine-tuning	1.9963038	WORD
6	verification	WORD,USER	multi-stage	1.9963038	WORD
**1980s.**					
**iterations**	**value**	**kind**	**word**	**score**	**kind2**
9	verification	WORD,USER	verification	8.141953	WORD
9	verification	WORD,USER	possesses	5.3206515	WORD
9	verification	WORD,USER	rrodels	4.9648595	WORD
9	verification	WORD,USER	entails	4.59189	WORD
9	verification	WORD,USER	formality	4.359849	WORD
9	verification	WORD,USER	furthering	4.359849	WORD
9	verification	WORD,USER	substantiating	4.359849	WORD
9	verification	WORD,USER	walk	4.359849	WORD
9	verification	WORD,USER	chamber	4.2061906	WORD
9	verification	WORD,USER	focussed	4.2061906	WORD
9	verification	WORD,USER	formulates	4.2061906	WORD
9	verification	WORD,USER	rrore	4.2061906	WORD
9	verification	WORD,USER	validates	4.2061906	WORD
9	verification	WORD,USER	breakpointing	4.008293	WORD
9	verification	WORD,USER	dtring	4.008293	WORD
9	verification	WORD,USER	faithful	4.008293	WORD
9	verification	WORD,USER	fielded	4.008293	WORD
9	verification	WORD,USER	non-simulators	4.008293	WORD
9	verification	WORD,USER	positivism	4.008293	WORD

9	verification	WORD,USER	prograrrrning	4.008293	WORD
9	verification	WORD,USER	sirrolation	4.008293	WORD
9	verification	WORD,USER	unclassified	4.008293	WORD
9	verification	WORD,USER	9ystems	3.7296562	WORD
9	verification	WORD,USER	absolve	3.7296562	WORD
9	verification	WORD,USER	accountability	3.7296562	WORD
9	verification	WORD,USER	activiti	3.7296562	WORD
9	verification	WORD,USER	anlayst	3.7296562	WORD
9	verification	WORD,USER	automata-theoretic	3.7296562	WORD
9	verification	WORD,USER	aver	3.7296562	WORD
9	verification	WORD,USER	behavior-mode	3.7296562	WORD
9	verification	WORD,USER	bolstering	3.7296562	WORD
9	verification	WORD,USER	categorically	3.7296562	WORD
9	verification	WORD,USER	claiming	3.7296562	WORD
9	verification	WORD,USER	cne	3.7296562	WORD
9	verification	WORD,USER	codify	3.7296562	WORD
9	verification	WORD,USER	colliding	3.7296562	WORD
9	verification	WORD,USER	computer-executable	3.7296562	WORD
9	verification	WORD,USER	conduc	3.7296562	WORD
9	verification	WORD,USER	conducive	3.7296562	WORD
9	verification	WORD,USER	confrontation	3.7296562	WORD
9	verification	WORD,USER	conitions	3.7296562	WORD
9	verification	WORD,USER	contaminate	3.7296562	WORD
9	verification	WORD,USER	correponding	3.7296562	WORD
9	verification	WORD,USER	corroborating	3.7296562	WORD
9	verification	WORD,USER	culminated	3.7296562	WORD
9	verification	WORD,USER	de&l	3.7296562	WORD
9	verification	WORD,USER	deception	3.7296562	WORD
9	verification	WORD,USER	delegate	3.7296562	WORD
9	verification	WORD,USER	dence	3.7296562	WORD
9	verification	WORD,USER	deougging	3.7296562	WORD
9	verification	WORD,USER	derated	3.7296562	WORD
9	verification	WORD,USER	descri	3.7296562	WORD
9	verification	WORD,USER	deta	3.7296562	WORD
9	verification	WORD,USER	developer's	3.7296562	WORD
9	verification	WORD,USER	dissect	3.7296562	WORD
9	verification	WORD,USER	elapses	3.7296562	WORD
9	verification	WORD,USER	empi	3.7296562	WORD

9	verification	WORD,USER	empiricists	3.7296562	WORD
9	verification	WORD,USER	ensured	3.7296562	WORD
9	verification	WORD,USER	entailing	3.7296562	WORD
9	verification	WORD,USER	epochs	3.7296562	WORD
9	verification	WORD,USER	equ	3.7296562	WORD
9	verification	WORD,USER	exper	3.7296562	WORD
9	verification	WORD,USER	falsificationist	3.7296562	WORD
9	verification	WORD,USER	films	3.7296562	WORD
9	verification	WORD,USER	firer-target	3.7296562	WORD
9	verification	WORD,USER	flowcharts	3.7296562	WORD
9	verification	WORD,USER	flowed	3.7296562	WORD
9	verification	WORD,USER	follbwing	3.7296562	WORD
9	verification	WORD,USER	ften	3.7296562	WORD
9	verification	WORD,USER	ges	3.7296562	WORD
9	verification	WORD,USER	gineering	3.7296562	WORD
9	verification	WORD,USER	hinted	3.7296562	WORD
9	verification	WORD,USER	histoqrams	3.7296562	WORD
9	verification	WORD,USER	historian	3.7296562	WORD
9	verification	WORD,USER	hurt	3.7296562	WORD
9	verification	WORD,USER	ilrooms	3.7296562	WORD
9	verification	WORD,USER	infonnally	3.7296562	WORD
9	verification	WORD,USER	infrequent	3.7296562	WORD
9	verification	WORD,USER	inp	3.7296562	WORD
9	verification	WORD,USER	instrumentation-based	3.7296562	WORD
9	verification	WORD,USER	involvenent	3.7296562	WORD
9	verification	WORD,USER	itate	3.7296562	WORD
9	verification	WORD,USER	lamda	3.7296562	WORD
9	verification	WORD,USER	linkers	3.7296562	WORD
9	verification	WORD,USER	logics	3.7296562	WORD
9	verification	WORD,USER	ltldeling	3.7296562	WORD
9	verification	WORD,USER	mathematica1	3.7296562	WORD
9	verification	WORD,USER	mixtures	3.7296562	WORD
9	verification	WORD,USER	model-building	3.7296562	WORD
9	verification	WORD,USER	moqel	3.7296562	WORD
9	verification	WORD,USER	neural	3.7296562	WORD
9	verification	WORD,USER	nical	3.7296562	WORD
9	verification	WORD,USER	nonsimulationists	3.7296562	WORD
9	verification	WORD,USER	oodel	3.7296562	WORD

9	verification	WORD,USER	oped	3.7296562	WORD
9	verification	WORD,USER	ophical	3.7296562	WORD
9	verification	WORD,USER	paraphrase	3.7296562	WORD
9	verification	WORD,USER	pulls	3.7296562	WORD
9	verification	WORD,USER	reap	3.7296562	WORD
9	verification	WORD,USER	reasonble	3.7296562	WORD
9	verification	WORD,USER	rectified	3.7296562	WORD
9	verification	WORD,USER	referee	3.7296562	WORD
9	verification	WORD,USER	reflexions	3.7296562	WORD
9	verification	WORD,USER	relegated	3.7296562	WORD
9	verification	WORD,USER	resent	3.7296562	WORD
9	verification	WORD,USER	retested	3.7296562	WORD
9	verification	WORD,USER	revolutions	3.7296562	WORD
9	verification	WORD,USER	rr1	3.7296562	WORD
9	verification	WORD,USER	rrost	3.7296562	WORD
9	verification	WORD,USER	sdb	3.7296562	WORD
9	verification	WORD,USER	seventies	3.7296562	WORD
9	verification	WORD,USER	shuffling	3.7296562	WORD
9	verification	WORD,USER	simulation-related	3.7296562	WORD
9	verification	WORD,USER	slavish	3.7296562	WORD
9	verification	WORD,USER	standalone	3.7296562	WORD
9	verification	WORD,USER	stemming	3.7296562	WORD
9	verification	WORD,USER	strengthened	3.7296562	WORD
9	verification	WORD,USER	striving	3.7296562	WORD
9	verification	WORD,USER	tci	3.7296562	WORD
9	verification	WORD,USER	therein	3.7296562	WORD
9	verification	WORD,USER	tide	3.7296562	WORD
9	verification	WORD,USER	touches	3.7296562	WORD
9	verification	WORD,USER	udy	3.7296562	WORD
9	verification	WORD,USER	ues	3.7296562	WORD
9	verification	WORD,USER	unt	3.7296562	WORD
9	verification	WORD,USER	walk-throughs	3.7296562	WORD
9	verification	WORD,USER	watches	3.7296562	WORD
9	verification	WORD,USER	wholly	3.7296562	WORD
9	verification	WORD,USER	wires	3.7296562	WORD
9	verification	WORD,USER	witli	3.7296562	WORD
9	verification	WORD,USER	instrumentalism	3.2538166	WORD
9	verification	WORD,USER	substantiation	3.0663395	WORD

9	verification	WORD,USER	continuation	2.9071023	WORD
9	verification	WORD,USER	echo	2.9071023	WORD
9	verification	WORD,USER	sinulation	2.8333216	WORD
9	verification	WORD,USER	reproducing	2.6277688	WORD
9	verification	WORD,USER	ensuring	2.6188927	WORD
9	verification	WORD,USER	ascertaining	2.4298708	WORD
9	verification	WORD,USER	categorizes	2.4298708	WORD
9	verification	WORD,USER	sh	2.4298708	WORD
9	verification	WORD,USER	tistical	2.4298708	WORD
9	verification	WORD,USER	stepping	2.3062618	WORD
9	verification	WORD,USER	practicality	2.1526036	WORD
9	verification	WORD,USER	rrodeling	2.1526036	WORD
9	verification	WORD,USER	adb	2.151234	WORD
9	verification	WORD,USER	defaulted	2.151234	WORD
9	verification	WORD,USER	undertake	2.151234	WORD
**1990s.**					
**iterations**	**value**	**kind**	**word**	**score**	**kind2**
10	verification	WORD,USER	verification	6.1748385	WORD
10	verification	WORD,USER	validation	6.130572	WORD
10	verification	WORD,USER	veri	5.9661365	WORD
10	verification	WORD,USER	assuring	5.073945	WORD
10	verification	WORD,USER	empiricism	4.861838	WORD
10	verification	WORD,USER	substantiation	4.861838	WORD
10	verification	WORD,USER	rationalism	4.775063	WORD
10	verification	WORD,USER	quicker	4.6767244	WORD
10	verification	WORD,USER	structure-oriented	4.6767244	WORD
10	verification	WORD,USER	substantiating	4.6767244	WORD
10	verification	WORD,USER	multi-step	4.563246	WORD
10	verification	WORD,USER	architects	4.4290814	WORD
10	verification	WORD,USER	classi	4.4290814	WORD
10	verification	WORD,USER	co-developed	4.4290814	WORD
10	verification	WORD,USER	impediments	4.4290814	WORD
10	verification	WORD,USER	instrumentalist	4.4290814	WORD
10	verification	WORD,USER	methodical	4.4290814	WORD
10	verification	WORD,USER	post-construction	4.4290814	WORD
10	verification	WORD,USER	reproducible	4.4290814	WORD

10	verification	WORD,USER	reprogramming	4.4290814	WORD
10	verification	WORD,USER	software-in-the-loop	4.4290814	WORD
10	verification	WORD,USER	approves	4.2649426	WORD
10	verification	WORD,USER	ascertaining	4.2649426	WORD
10	verification	WORD,USER	culminated	4.2649426	WORD
10	verification	WORD,USER	idealised	4.2649426	WORD
10	verification	WORD,USER	inconvenient	4.2649426	WORD
10	verification	WORD,USER	meso	4.2649426	WORD
10	verification	WORD,USER	cation	4.259617	WORD
10	verification	WORD,USER	analysiddesign	4.053414	WORD
10	verification	WORD,USER	certi	4.053414	WORD
10	verification	WORD,USER	confederations	4.053414	WORD
10	verification	WORD,USER	confrontational	4.053414	WORD
10	verification	WORD,USER	conventionalistic	4.053414	WORD
10	verification	WORD,USER	correspondences	4.053414	WORD
10	verification	WORD,USER	critics	4.053414	WORD
10	verification	WORD,USER	critiques	4.053414	WORD
10	verification	WORD,USER	direct-structure	4.053414	WORD
10	verification	WORD,USER	dismissed	4.053414	WORD
10	verification	WORD,USER	hasten	4.053414	WORD
10	verification	WORD,USER	illuminates	4.053414	WORD
10	verification	WORD,USER	informality	4.053414	WORD
10	verification	WORD,USER	inhibit	4.053414	WORD
10	verification	WORD,USER	initiator	4.053414	WORD
10	verification	WORD,USER	man-in	4.053414	WORD
10	verification	WORD,USER	model-test-model	4.053414	WORD
10	verification	WORD,USER	post-condition	4.053414	WORD
10	verification	WORD,USER	refutation	4.053414	WORD
10	verification	WORD,USER	regressing	4.053414	WORD
10	verification	WORD,USER	revalidated	4.053414	WORD
10	verification	WORD,USER	revalidations	4.053414	WORD
10	verification	WORD,USER	revolutionized	4.053414	WORD
10	verification	WORD,USER	simulation-model	4.053414	WORD
10	verification	WORD,USER	specificity	4.053414	WORD
10	verification	WORD,USER	strengthens	4.053414	WORD
10	verification	WORD,USER	suites	4.053414	WORD
10	verification	WORD,USER	undocumented	4.053414	WORD
10	verification	WORD,USER	vee	4.053414	WORD

10	verification	WORD,USER	whisker	4.053414	WORD
10	verification	WORD,USER	wildly	4.053414	WORD
10	verification	WORD,USER	absolutist	3.7553992	WORD
10	verification	WORD,USER	advises	3.7553992	WORD
10	verification	WORD,USER	alternati	3.7553992	WORD
10	verification	WORD,USER	audits	3.7553992	WORD
10	verification	WORD,USER	beta-testing	3.7553992	WORD
10	verification	WORD,USER	bore	3.7553992	WORD
10	verification	WORD,USER	byproduct	3.7553992	WORD
10	verification	WORD,USER	clarifies	3.7553992	WORD
10	verification	WORD,USER	color-coded	3.7553992	WORD
10	verification	WORD,USER	compositional	3.7553992	WORD
10	verification	WORD,USER	crowds	3.7553992	WORD
10	verification	WORD,USER	data-input	3.7553992	WORD
10	verification	WORD,USER	deductions	3.7553992	WORD
10	verification	WORD,USER	eigenvector	3.7553992	WORD
10	verification	WORD,USER	emitters	3.7553992	WORD
10	verification	WORD,USER	etal	3.7553992	WORD
10	verification	WORD,USER	fears	3.7553992	WORD
10	verification	WORD,USER	footnotes	3.7553992	WORD
10	verification	WORD,USER	formalising	3.7553992	WORD
10	verification	WORD,USER	ground-truth	3.7553992	WORD
10	verification	WORD,USER	holdings	3.7553992	WORD
10	verification	WORD,USER	hole	3.7553992	WORD
10	verification	WORD,USER	illogical	3.7553992	WORD
10	verification	WORD,USER	initialisation	3.7553992	WORD
10	verification	WORD,USER	institutionalizes	3.7553992	WORD
10	verification	WORD,USER	intermingled	3.7553992	WORD
10	verification	WORD,USER	knowledgebase	3.7553992	WORD
10	verification	WORD,USER	liberally	3.7553992	WORD
10	verification	WORD,USER	meaningfulness	3.7553992	WORD
10	verification	WORD,USER	methodically	3.7553992	WORD
10	verification	WORD,USER	mimicking	3.7553992	WORD
10	verification	WORD,USER	morphological	3.7553992	WORD
10	verification	WORD,USER	myths	3.7553992	WORD
10	verification	WORD,USER	non-modelers	3.7553992	WORD
10	verification	WORD,USER	positivism	3.7553992	WORD
10	verification	WORD,USER	pre-emption	3.7553992	WORD

10	verification	WORD,USER	pretest	3.7553992	WORD
10	verification	WORD,USER	producer's	3.7553992	WORD
10	verification	WORD,USER	project’s	3.7553992	WORD
10	verification	WORD,USER	quadrant	3.7553992	WORD
10	verification	WORD,USER	sideby-side	3.7553992	WORD
10	verification	WORD,USER	specification-oriented	3.7553992	WORD
10	verification	WORD,USER	study’s	3.7553992	WORD
10	verification	WORD,USER	subjecting	3.7553992	WORD
10	verification	WORD,USER	systolic	3.7553992	WORD
10	verification	WORD,USER	tantamount	3.7553992	WORD
10	verification	WORD,USER	umbrella	3.7553992	WORD
10	verification	WORD,USER	uneconomical	3.7553992	WORD
10	verification	WORD,USER	unproductive	3.7553992	WORD
10	verification	WORD,USER	unvalidatable	3.7553992	WORD
10	verification	WORD,USER	visually-based	3.7553992	WORD
10	verification	WORD,USER	write-up	3.7553992	WORD
10	verification	WORD,USER	σy	3.7553992	WORD
10	verification	WORD,USER	σω	3.7553992	WORD
10	verification	WORD,USER	verifiable	3.2487233	WORD
10	verification	WORD,USER	abreast	3.2461436	WORD
10	verification	WORD,USER	authentication	3.2461436	WORD
10	verification	WORD,USER	battlefields	3.2461436	WORD
10	verification	WORD,USER	behavior’s	3.2461436	WORD
10	verification	WORD,USER	blends	3.2461436	WORD
10	verification	WORD,USER	bug-free	3.2461436	WORD
10	verification	WORD,USER	checkers	3.2461436	WORD
10	verification	WORD,USER	co-chairs	3.2461436	WORD
10	verification	WORD,USER	complexes	3.2461436	WORD
10	verification	WORD,USER	conceptualise	3.2461436	WORD
10	verification	WORD,USER	confinn	3.2461436	WORD
10	verification	WORD,USER	contended	3.2461436	WORD
10	verification	WORD,USER	cross-validation	3.2461436	WORD
10	verification	WORD,USER	day’s	3.2461436	WORD
10	verification	WORD,USER	decisively	3.2461436	WORD
10	verification	WORD,USER	descending	3.2461436	WORD
10	verification	WORD,USER	deteriorated	3.2461436	WORD
10	verification	WORD,USER	deviated	3.2461436	WORD
10	verification	WORD,USER	divergences	3.2461436	WORD

10	verification	WORD,USER	easy-to-understand	3.2461436	WORD
10	verification	WORD,USER	fulfils	3.2461436	WORD
10	verification	WORD,USER	grading	3.2461436	WORD
10	verification	WORD,USER	hears	3.2461436	WORD
10	verification	WORD,USER	holdout	3.2461436	WORD
10	verification	WORD,USER	hopeful	3.2461436	WORD
10	verification	WORD,USER	idation	3.2461436	WORD
10	verification	WORD,USER	inadequacy	3.2461436	WORD
10	verification	WORD,USER	incumbent	3.2461436	WORD
10	verification	WORD,USER	laborious	3.2461436	WORD
10	verification	WORD,USER	lamented	3.2461436	WORD
10	verification	WORD,USER	loses	3.2461436	WORD
10	verification	WORD,USER	manageability	3.2461436	WORD
10	verification	WORD,USER	meticulous	3.2461436	WORD
10	verification	WORD,USER	mirrored	3.2461436	WORD
10	verification	WORD,USER	moderators	3.2461436	WORD
10	verification	WORD,USER	mph	3.2461436	WORD
10	verification	WORD,USER	mutation	3.2461436	WORD
10	verification	WORD,USER	non-native	3.2461436	WORD
10	verification	WORD,USER	notoriously	3.2461436	WORD
10	verification	WORD,USER	organization’s	3.2461436	WORD
10	verification	WORD,USER	organizer	3.2461436	WORD
10	verification	WORD,USER	owchart	3.2461436	WORD
10	verification	WORD,USER	patrolling	3.2461436	WORD
10	verification	WORD,USER	peculiarities	3.2461436	WORD
10	verification	WORD,USER	perspicuity	3.2461436	WORD
10	verification	WORD,USER	plethora	3.2461436	WORD
10	verification	WORD,USER	pre-specified	3.2461436	WORD
10	verification	WORD,USER	primacy	3.2461436	WORD
10	verification	WORD,USER	reaped	3.2461436	WORD
10	verification	WORD,USER	regressions	3.2461436	WORD
10	verification	WORD,USER	repairman	3.2461436	WORD
10	verification	WORD,USER	self-similarity	3.2461436	WORD
10	verification	WORD,USER	shortens	3.2461436	WORD
10	verification	WORD,USER	signal-to-noise	3.2461436	WORD
10	verification	WORD,USER	situation-specific	3.2461436	WORD
10	verification	WORD,USER	soul	3.2461436	WORD
10	verification	WORD,USER	stakeholder’s	3.2461436	WORD

10	verification	WORD,USER	standards-based	3.2461436	WORD
10	verification	WORD,USER	t-statistic	3.2461436	WORD
10	verification	WORD,USER	talces	3.2461436	WORD
10	verification	WORD,USER	the-loop	3.2461436	WORD
10	verification	WORD,USER	toplevel	3.2461436	WORD
10	verification	WORD,USER	triumph	3.2461436	WORD
10	verification	WORD,USER	two-man	3.2461436	WORD
10	verification	WORD,USER	unachievable	3.2461436	WORD
10	verification	WORD,USER	underwent	3.2461436	WORD
10	verification	WORD,USER	user-controlled	3.2461436	WORD
10	verification	WORD,USER	v1	3.2461436	WORD
10	verification	WORD,USER	variability-sensitivity	3.2461436	WORD
10	verification	WORD,USER	wary	3.2461436	WORD
10	verification	WORD,USER	ˇ	3.2461436	WORD
10	verification	WORD,USER	schemas	2.872478	WORD
10	verification	WORD,USER	surveyed	2.7383137	WORD
10	verification	WORD,USER	multistage	2.5764554	WORD
10	verification	WORD,USER	endless	2.574175	WORD
10	verification	WORD,USER	gleaned	2.574175	WORD
10	verification	WORD,USER	mediation	2.574175	WORD
10	verification	WORD,USER	plausibility	2.362646	WORD
10	verification	WORD,USER	complied	2.0646312	WORD
10	verification	WORD,USER	complimentary	2.0646312	WORD
10	verification	WORD,USER	sealing	2.0646312	WORD
10	verification	WORD,USER	sensation	2.0646312	WORD
10	verification	WORD,USER	unintentional	2.0646312	WORD
10	verification	WORD,USER	prolonged	1.853665	WORD
**2000s.**					
**iterations**	**value**	**kind**	**word**	**score**	**kind2**
9	verification	WORD,USER	validation	6.136519	WORD
9	verification	WORD,USER	verification	6.039942	WORD
9	verification	WORD,USER	contingent	4.750617	WORD
9	verification	WORD,USER	walkthroughs	4.5700154	WORD
9	verification	WORD,USER	ascertaining	4.459303	WORD
9	verification	WORD,USER	formalisation	4.459303	WORD
9	verification	WORD,USER	gradebreaks	4.459303	WORD

9	verification	WORD,USER	revalidation	4.459303	WORD
9	verification	WORD,USER	intentionality	4.32841	WORD
9	verification	WORD,USER	crosswalk	4.1682734	WORD
9	verification	WORD,USER	illuminates	4.1682734	WORD
9	verification	WORD,USER	reprogramming	4.1682734	WORD
9	verification	WORD,USER	toplevel	4.1682734	WORD
9	verification	WORD,USER	verity	4.1682734	WORD
9	verification	WORD,USER	conceptual-model	3.961904	WORD
9	verification	WORD,USER	confidences	3.961904	WORD
9	verification	WORD,USER	data-poor	3.961904	WORD
9	verification	WORD,USER	data-rich	3.961904	WORD
9	verification	WORD,USER	descends	3.961904	WORD
9	verification	WORD,USER	discriminant	3.961904	WORD
9	verification	WORD,USER	empiricism	3.961904	WORD
9	verification	WORD,USER	empiricist	3.961904	WORD
9	verification	WORD,USER	generalizable	3.961904	WORD
9	verification	WORD,USER	knowledgebased	3.961904	WORD
9	verification	WORD,USER	meta-component	3.961904	WORD
9	verification	WORD,USER	non-statistical	3.961904	WORD
9	verification	WORD,USER	provenance	3.961904	WORD
9	verification	WORD,USER	rationalism	3.961904	WORD
9	verification	WORD,USER	rationalist	3.961904	WORD
9	verification	WORD,USER	relativist	3.961904	WORD
9	verification	WORD,USER	scrupulous	3.961904	WORD
9	verification	WORD,USER	truthfulness	3.961904	WORD
9	verification	WORD,USER	accreditations	3.6711588	WORD
9	verification	WORD,USER	addendum	3.6711588	WORD
9	verification	WORD,USER	agreeing	3.6711588	WORD
9	verification	WORD,USER	air-traffic	3.6711588	WORD
9	verification	WORD,USER	allergic	3.6711588	WORD
9	verification	WORD,USER	breakpoint	3.6711588	WORD
9	verification	WORD,USER	bulletin	3.6711588	WORD
9	verification	WORD,USER	co-exist	3.6711588	WORD
9	verification	WORD,USER	coder	3.6711588	WORD
9	verification	WORD,USER	communiqués	3.6711588	WORD
9	verification	WORD,USER	comprehendible	3.6711588	WORD
9	verification	WORD,USER	compressed	3.6711588	WORD
9	verification	WORD,USER	compressing	3.6711588	WORD

9	verification	WORD,USER	contemporaneous	3.6711588	WORD
9	verification	WORD,USER	devel	3.6711588	WORD
9	verification	WORD,USER	diligent	3.6711588	WORD
9	verification	WORD,USER	disaggregating	3.6711588	WORD
9	verification	WORD,USER	documentary	3.6711588	WORD
9	verification	WORD,USER	domain-experts	3.6711588	WORD
9	verification	WORD,USER	els	3.6711588	WORD
9	verification	WORD,USER	evaluative	3.6711588	WORD
9	verification	WORD,USER	explicit-state	3.6711588	WORD
9	verification	WORD,USER	expository	3.6711588	WORD
9	verification	WORD,USER	extrema	3.6711588	WORD
9	verification	WORD,USER	extreme-conditions	3.6711588	WORD
9	verification	WORD,USER	finalizing	3.6711588	WORD
9	verification	WORD,USER	foundationalist	3.6711588	WORD
9	verification	WORD,USER	holographic	3.6711588	WORD
9	verification	WORD,USER	in-class	3.6711588	WORD
9	verification	WORD,USER	in-place	3.6711588	WORD
9	verification	WORD,USER	keying	3.6711588	WORD
9	verification	WORD,USER	lls	3.6711588	WORD
9	verification	WORD,USER	machine-readable	3.6711588	WORD
9	verification	WORD,USER	makefiles	3.6711588	WORD
9	verification	WORD,USER	most-important	3.6711588	WORD
9	verification	WORD,USER	multi-processor	3.6711588	WORD
9	verification	WORD,USER	navigated	3.6711588	WORD
9	verification	WORD,USER	negating	3.6711588	WORD
9	verification	WORD,USER	nonexistent	3.6711588	WORD
9	verification	WORD,USER	onus	3.6711588	WORD
9	verification	WORD,USER	organization's	3.6711588	WORD
9	verification	WORD,USER	ply	3.6711588	WORD
9	verification	WORD,USER	programmatics	3.6711588	WORD
9	verification	WORD,USER	quotes	3.6711588	WORD
9	verification	WORD,USER	redactor	3.6711588	WORD
9	verification	WORD,USER	researcher's	3.6711588	WORD
9	verification	WORD,USER	ripple	3.6711588	WORD
9	verification	WORD,USER	seasoned	3.6711588	WORD
9	verification	WORD,USER	software-in-the-loop	3.6711588	WORD
9	verification	WORD,USER	specification-calibration	3.6711588	WORD
9	verification	WORD,USER	standardizes	3.6711588	WORD

9	verification	WORD,USER	sub-modules	3.6711588	WORD
9	verification	WORD,USER	synthers	3.6711588	WORD
9	verification	WORD,USER	table-based	3.6711588	WORD
9	verification	WORD,USER	tampering	3.6711588	WORD
9	verification	WORD,USER	teamed	3.6711588	WORD
9	verification	WORD,USER	terabyte	3.6711588	WORD
9	verification	WORD,USER	time-flow	3.6711588	WORD
9	verification	WORD,USER	tool-set	3.6711588	WORD
9	verification	WORD,USER	touches	3.6711588	WORD
9	verification	WORD,USER	transacted	3.6711588	WORD
9	verification	WORD,USER	unsure	3.6711588	WORD
9	verification	WORD,USER	unsustainable	3.6711588	WORD
9	verification	WORD,USER	variability-sensitivity	3.6711588	WORD
9	verification	WORD,USER	advises	3.1743276	WORD
9	verification	WORD,USER	alleged	3.1743276	WORD
9	verification	WORD,USER	alternated	3.1743276	WORD
9	verification	WORD,USER	amalgamation	3.1743276	WORD
9	verification	WORD,USER	anchors	3.1743276	WORD
9	verification	WORD,USER	andre	3.1743276	WORD
9	verification	WORD,USER	archaeological	3.1743276	WORD
9	verification	WORD,USER	automaticall	3.1743276	WORD
9	verification	WORD,USER	back-propagation	3.1743276	WORD
9	verification	WORD,USER	battlespaces	3.1743276	WORD
9	verification	WORD,USER	behavior-sensitivity	3.1743276	WORD
9	verification	WORD,USER	bhr	3.1743276	WORD
9	verification	WORD,USER	binaries	3.1743276	WORD
9	verification	WORD,USER	boxing	3.1743276	WORD
9	verification	WORD,USER	cementing	3.1743276	WORD
9	verification	WORD,USER	cha	3.1743276	WORD
9	verification	WORD,USER	citations	3.1743276	WORD
9	verification	WORD,USER	clearness	3.1743276	WORD
9	verification	WORD,USER	co-chaired	3.1743276	WORD
9	verification	WORD,USER	cognizance	3.1743276	WORD
9	verification	WORD,USER	collectable	3.1743276	WORD
9	verification	WORD,USER	combats	3.1743276	WORD
9	verification	WORD,USER	commercialization	3.1743276	WORD
9	verification	WORD,USER	computerization	3.1743276	WORD
9	verification	WORD,USER	conflated	3.1743276	WORD

9	verification	WORD,USER	conversational	3.1743276	WORD
9	verification	WORD,USER	corroborate	3.1743276	WORD
9	verification	WORD,USER	cost-risk	3.1743276	WORD
9	verification	WORD,USER	costeffectiveness	3.1743276	WORD
9	verification	WORD,USER	costrisk	3.1743276	WORD
9	verification	WORD,USER	counter-part	3.1743276	WORD
9	verification	WORD,USER	cross-element	3.1743276	WORD
9	verification	WORD,USER	cross-model	3.1743276	WORD
9	verification	WORD,USER	decidedly	3.1743276	WORD
9	verification	WORD,USER	deepening	3.1743276	WORD
9	verification	WORD,USER	dential	3.1743276	WORD
9	verification	WORD,USER	developerworks	3.1743276	WORD
9	verification	WORD,USER	discriminated	3.1743276	WORD
9	verification	WORD,USER	distillation	3.1743276	WORD
9	verification	WORD,USER	diversely	3.1743276	WORD
9	verification	WORD,USER	docu	3.1743276	WORD
9	verification	WORD,USER	double-pulse	3.1743276	WORD
9	verification	WORD,USER	early-stage	3.1743276	WORD
9	verification	WORD,USER	embarrassment	3.1743276	WORD
9	verification	WORD,USER	empirical-strong	3.1743276	WORD
9	verification	WORD,USER	empirical-weak	3.1743276	WORD
9	verification	WORD,USER	extrapolations	3.1743276	WORD
9	verification	WORD,USER	factorization	3.1743276	WORD
9	verification	WORD,USER	ff	3.1743276	WORD
9	verification	WORD,USER	fies	3.1743276	WORD
9	verification	WORD,USER	furthering	3.1743276	WORD
9	verification	WORD,USER	garrison	3.1743276	WORD
9	verification	WORD,USER	girder	3.1743276	WORD
9	verification	WORD,USER	government-supported	3.1743276	WORD
9	verification	WORD,USER	graded	3.1743276	WORD
9	verification	WORD,USER	guidebook	3.1743276	WORD
9	verification	WORD,USER	hyperbolic	3.1743276	WORD
9	verification	WORD,USER	inanimate	3.1743276	WORD
9	verification	WORD,USER	inexactness	3.1743276	WORD
9	verification	WORD,USER	ings	3.1743276	WORD
9	verification	WORD,USER	insects	3.1743276	WORD
9	verification	WORD,USER	interoperated	3.1743276	WORD
9	verification	WORD,USER	investor	3.1743276	WORD

9	verification	WORD,USER	kilobytes	3.1743276	WORD
9	verification	WORD,USER	levies	3.1743276	WORD
9	verification	WORD,USER	location-aware	3.1743276	WORD
9	verification	WORD,USER	loom	3.1743276	WORD
9	verification	WORD,USER	materialhandling	3.1743276	WORD
9	verification	WORD,USER	member-task	3.1743276	WORD
9	verification	WORD,USER	micro-world	3.1743276	WORD
9	verification	WORD,USER	microhydro	3.1743276	WORD
9	verification	WORD,USER	misconception	3.1743276	WORD
9	verification	WORD,USER	mobilized	3.1743276	WORD
9	verification	WORD,USER	model-making	3.1743276	WORD
9	verification	WORD,USER	nickname	3.1743276	WORD
9	verification	WORD,USER	non-repeatable	3.1743276	WORD
9	verification	WORD,USER	notoriously	3.1743276	WORD
9	verification	WORD,USER	participatory-based	3.1743276	WORD
9	verification	WORD,USER	persuaded	3.1743276	WORD
9	verification	WORD,USER	pervades	3.1743276	WORD
9	verification	WORD,USER	philosopher	3.1743276	WORD
9	verification	WORD,USER	planing	3.1743276	WORD
9	verification	WORD,USER	polyline	3.1743276	WORD
9	verification	WORD,USER	postmortem	3.1743276	WORD
9	verification	WORD,USER	praised	3.1743276	WORD
9	verification	WORD,USER	preoccupation	3.1743276	WORD
9	verification	WORD,USER	quietly	3.1743276	WORD
9	verification	WORD,USER	real-system	3.1743276	WORD
9	verification	WORD,USER	recertification	3.1743276	WORD
9	verification	WORD,USER	regaining	3.1743276	WORD
9	verification	WORD,USER	releasable	3.1743276	WORD
9	verification	WORD,USER	retirements	3.1743276	WORD
9	verification	WORD,USER	reuser	3.1743276	WORD
9	verification	WORD,USER	sanctioning	3.1743276	WORD
9	verification	WORD,USER	sandpapering	3.1743276	WORD
9	verification	WORD,USER	seg	3.1743276	WORD
9	verification	WORD,USER	semantical	3.1743276	WORD
9	verification	WORD,USER	semi-formal	3.1743276	WORD
9	verification	WORD,USER	specialisations	3.1743276	WORD
9	verification	WORD,USER	speculated	3.1743276	WORD
9	verification	WORD,USER	spin-off	3.1743276	WORD

9	verification	WORD,USER	standpoints	3.1743276	WORD
9	verification	WORD,USER	starter	3.1743276	WORD
9	verification	WORD,USER	structural-strong	3.1743276	WORD
9	verification	WORD,USER	structure-oriented	3.1743276	WORD
9	verification	WORD,USER	supposition	3.1743276	WORD
9	verification	WORD,USER	surrounds	3.1743276	WORD
9	verification	WORD,USER	system-on-chip	3.1743276	WORD
9	verification	WORD,USER	tamper-detecting	3.1743276	WORD
9	verification	WORD,USER	to-be	3.1743276	WORD
9	verification	WORD,USER	triangulating	3.1743276	WORD
9	verification	WORD,USER	trusting	3.1743276	WORD
9	verification	WORD,USER	typographic	3.1743276	WORD
9	verification	WORD,USER	user-adaptable	3.1743276	WORD
9	verification	WORD,USER	verbalise	3.1743276	WORD
9	verification	WORD,USER	verifier	3.1743276	WORD
9	verification	WORD,USER	voids	3.1743276	WORD
9	verification	WORD,USER	waived	3.1743276	WORD
9	verification	WORD,USER	wine	3.1743276	WORD
9	verification	WORD,USER	ðn	3.1743276	WORD
9	verification	WORD,USER	two-sample	2.8097959	WORD
9	verification	WORD,USER	project's	2.5187664	WORD
9	verification	WORD,USER	human-machine	2.312397	WORD
9	verification	WORD,USER	earnest	2.0216517	WORD
9	verification	WORD,USER	misuse	2.0216517	WORD
9	verification	WORD,USER	onsite	2.0216517	WORD
9	verification	WORD,USER	unstated	2.0216517	WORD

2010s.

iterations	value	kind	word	score	kind2
9	verification	WORD,USER	verification	7.5837445	WORD
9	verification	WORD,USER	accreditation	6.0865097	WORD
9	verification	WORD,USER	statechart-assertions	4.354145	WORD
9	verification	WORD,USER	respondent	4.234495	WORD
9	verification	WORD,USER	definitively	4.088172	WORD
9	verification	WORD,USER	time-constrained	4.088172	WORD
9	verification	WORD,USER	35th	3.8996813	WORD
9	verification	WORD,USER	afterthought	3.8996813	WORD

9	verification	WORD,USER	apt	3.8996813	WORD
9	verification	WORD,USER	culminates	3.8996813	WORD
9	verification	WORD,USER	darker	3.8996813	WORD
9	verification	WORD,USER	demise	3.8996813	WORD
9	verification	WORD,USER	endgame	3.8996813	WORD
9	verification	WORD,USER	entailment	3.8996813	WORD
9	verification	WORD,USER	equaled	3.8996813	WORD
9	verification	WORD,USER	execution-based	3.8996813	WORD
9	verification	WORD,USER	explainable	3.8996813	WORD
9	verification	WORD,USER	hides	3.8996813	WORD
9	verification	WORD,USER	inter-agent	3.8996813	WORD
9	verification	WORD,USER	locational	3.8996813	WORD
9	verification	WORD,USER	mastering	3.8996813	WORD
9	verification	WORD,USER	matrixes	3.8996813	WORD
9	verification	WORD,USER	motive	3.8996813	WORD
9	verification	WORD,USER	non-bottlenecks	3.8996813	WORD
9	verification	WORD,USER	outweighed	3.8996813	WORD
9	verification	WORD,USER	parameterisation	3.8996813	WORD
9	verification	WORD,USER	proficient	3.8996813	WORD
9	verification	WORD,USER	qua	3.8996813	WORD
9	verification	WORD,USER	re-create	3.8996813	WORD
9	verification	WORD,USER	restarted	3.8996813	WORD
9	verification	WORD,USER	specializes	3.8996813	WORD
9	verification	WORD,USER	statechartassertions	3.8996813	WORD
9	verification	WORD,USER	stolen	3.8996813	WORD
9	verification	WORD,USER	stressful	3.8996813	WORD
9	verification	WORD,USER	tenuous	3.8996813	WORD
9	verification	WORD,USER	21th	3.6342323	WORD
9	verification	WORD,USER	36th	3.6342323	WORD
9	verification	WORD,USER	abduction	3.6342323	WORD
9	verification	WORD,USER	common-sense	3.6342323	WORD
9	verification	WORD,USER	critic	3.6342323	WORD
9	verification	WORD,USER	crunch	3.6342323	WORD
9	verification	WORD,USER	delve	3.6342323	WORD
9	verification	WORD,USER	elaborations	3.6342323	WORD
9	verification	WORD,USER	encumbered	3.6342323	WORD
9	verification	WORD,USER	endoscopic	3.6342323	WORD
9	verification	WORD,USER	evidence-driven	3.6342323	WORD

9	verification	WORD,USER	examin	3.6342323	WORD
9	verification	WORD,USER	excerpts	3.6342323	WORD
9	verification	WORD,USER	expressible	3.6342323	WORD
9	verification	WORD,USER	facial	3.6342323	WORD
9	verification	WORD,USER	flowtime	3.6342323	WORD
9	verification	WORD,USER	foray	3.6342323	WORD
9	verification	WORD,USER	fraught	3.6342323	WORD
9	verification	WORD,USER	fullest	3.6342323	WORD
9	verification	WORD,USER	grammars	3.6342323	WORD
9	verification	WORD,USER	guise	3.6342323	WORD
9	verification	WORD,USER	hig	3.6342323	WORD
9	verification	WORD,USER	human-induced	3.6342323	WORD
9	verification	WORD,USER	impediment	3.6342323	WORD
9	verification	WORD,USER	implausible	3.6342323	WORD
9	verification	WORD,USER	inaccessible	3.6342323	WORD
9	verification	WORD,USER	inexpensively	3.6342323	WORD
9	verification	WORD,USER	inscriptions	3.6342323	WORD
9	verification	WORD,USER	inter-system	3.6342323	WORD
9	verification	WORD,USER	interviewer	3.6342323	WORD
9	verification	WORD,USER	literal	3.6342323	WORD
9	verification	WORD,USER	lunches	3.6342323	WORD
9	verification	WORD,USER	methodical	3.6342323	WORD
9	verification	WORD,USER	negated	3.6342323	WORD
9	verification	WORD,USER	nnection	3.6342323	WORD
9	verification	WORD,USER	non-simulation	3.6342323	WORD
9	verification	WORD,USER	out-of-sample	3.6342323	WORD
9	verification	WORD,USER	painstaking	3.6342323	WORD
9	verification	WORD,USER	persisting	3.6342323	WORD
9	verification	WORD,USER	precursors	3.6342323	WORD
9	verification	WORD,USER	preparers	3.6342323	WORD
9	verification	WORD,USER	preselected	3.6342323	WORD
9	verification	WORD,USER	professions	3.6342323	WORD
9	verification	WORD,USER	profitably	3.6342323	WORD
9	verification	WORD,USER	quickness	3.6342323	WORD
9	verification	WORD,USER	refugees	3.6342323	WORD
9	verification	WORD,USER	rumor	3.6342323	WORD
9	verification	WORD,USER	rumors	3.6342323	WORD
9	verification	WORD,USER	stepby-step	3.6342323	WORD

9	verification	WORD,USER	subject-matter	3.6342323	WORD
9	verification	WORD,USER	superposed	3.6342323	WORD
9	verification	WORD,USER	systemof-systems	3.6342323	WORD
9	verification	WORD,USER	theo	3.6342323	WORD
9	verification	WORD,USER	threatened	3.6342323	WORD
9	verification	WORD,USER	tremendously	3.6342323	WORD
9	verification	WORD,USER	unaltered	3.6342323	WORD
9	verification	WORD,USER	unauthorized	3.6342323	WORD
9	verification	WORD,USER	undue	3.6342323	WORD
9	verification	WORD,USER	unverified	3.6342323	WORD
9	verification	WORD,USER	wife	3.6342323	WORD
9	verification	WORD,USER	wound	3.6342323	WORD
9	verification	WORD,USER	ablation	3.1808145	WORD
9	verification	WORD,USER	advantageously	3.1808145	WORD
9	verification	WORD,USER	airway	3.1808145	WORD
9	verification	WORD,USER	alidation	3.1808145	WORD
9	verification	WORD,USER	alysis	3.1808145	WORD
9	verification	WORD,USER	anal	3.1808145	WORD
9	verification	WORD,USER	ansson	3.1808145	WORD
9	verification	WORD,USER	archaeology	3.1808145	WORD
9	verification	WORD,USER	argmax	3.1808145	WORD
9	verification	WORD,USER	axiom	3.1808145	WORD
9	verification	WORD,USER	beginner	3.1808145	WORD
9	verification	WORD,USER	bleeding	3.1808145	WORD
9	verification	WORD,USER	boys	3.1808145	WORD
9	verification	WORD,USER	cadaver	3.1808145	WORD
9	verification	WORD,USER	case-studies	3.1808145	WORD
9	verification	WORD,USER	circumvent	3.1808145	WORD
9	verification	WORD,USER	clashes	3.1808145	WORD
9	verification	WORD,USER	codecs	3.1808145	WORD
9	verification	WORD,USER	code’s	3.1808145	WORD
9	verification	WORD,USER	community’s	3.1808145	WORD
9	verification	WORD,USER	computerised	3.1808145	WORD
9	verification	WORD,USER	decision-oriented	3.1808145	WORD
9	verification	WORD,USER	designtime	3.1808145	WORD
9	verification	WORD,USER	drill-down	3.1808145	WORD
9	verification	WORD,USER	du	3.1808145	WORD
9	verification	WORD,USER	eldwork	3.1808145	WORD

9	verification	WORD,USER	excuse	3.1808145	WORD
9	verification	WORD,USER	fax	3.1808145	WORD
9	verification	WORD,USER	form-finding	3.1808145	WORD
9	verification	WORD,USER	gh	3.1808145	WORD
9	verification	WORD,USER	guidewords	3.1808145	WORD
9	verification	WORD,USER	heavyweight	3.1808145	WORD
9	verification	WORD,USER	heel	3.1808145	WORD
9	verification	WORD,USER	humble	3.1808145	WORD
9	verification	WORD,USER	hungry	3.1808145	WORD
9	verification	WORD,USER	illuminates	3.1808145	WORD
9	verification	WORD,USER	intra-agent	3.1808145	WORD
9	verification	WORD,USER	inverting	3.1808145	WORD
9	verification	WORD,USER	minimalist	3.1808145	WORD
9	verification	WORD,USER	mock-ups	3.1808145	WORD
9	verification	WORD,USER	modifica	3.1808145	WORD
9	verification	WORD,USER	ngineering	3.1808145	WORD
9	verification	WORD,USER	non-ground	3.1808145	WORD
9	verification	WORD,USER	obstruction	3.1808145	WORD
9	verification	WORD,USER	onveyor	3.1808145	WORD
9	verification	WORD,USER	ories	3.1808145	WORD
9	verification	WORD,USER	p-t	3.1808145	WORD
9	verification	WORD,USER	person's	3.1808145	WORD
9	verification	WORD,USER	prefabrication	3.1808145	WORD
9	verification	WORD,USER	prospected	3.1808145	WORD
9	verification	WORD,USER	pubs	3.1808145	WORD
9	verification	WORD,USER	raining	3.1808145	WORD
9	verification	WORD,USER	re-implementing	3.1808145	WORD
9	verification	WORD,USER	recreates	3.1808145	WORD
9	verification	WORD,USER	retractions	3.1808145	WORD
9	verification	WORD,USER	revalidated	3.1808145	WORD
9	verification	WORD,USER	satisfiability	3.1808145	WORD
9	verification	WORD,USER	scale-model	3.1808145	WORD
9	verification	WORD,USER	sectarian	3.1808145	WORD
9	verification	WORD,USER	shining	3.1808145	WORD
9	verification	WORD,USER	simulati	3.1808145	WORD
9	verification	WORD,USER	ster	3.1808145	WORD
9	verification	WORD,USER	tan	3.1808145	WORD
9	verification	WORD,USER	testament	3.1808145	WORD

9	verification	WORD,USER	theaters	3.1808145	WORD
9	verification	WORD,USER	trace-driven	3.1808145	WORD
9	verification	WORD,USER	trusts	3.1808145	WORD
9	verification	WORD,USER	upper-level	3.1808145	WORD
9	verification	WORD,USER	ust	3.1808145	WORD
9	verification	WORD,USER	veterans	3.1808145	WORD
9	verification	WORD,USER	well-studied	3.1808145	WORD
9	verification	WORD,USER	verifiable	2.9508884	WORD
9	verification	WORD,USER	cognitively	2.8496442	WORD
9	verification	WORD,USER	rightly	2.729994	WORD
9	verification	WORD,USER	substantiation	2.729994	WORD
9	verification	WORD,USER	enlightening	2.5836713	WORD
9	verification	WORD,USER	near-term	2.5836713	WORD
9	verification	WORD,USER	testers	2.39518	WORD
9	verification	WORD,USER	validations	2.39518	WORD
9	verification	WORD,USER	zsignal	2.39518	WORD
9	verification	WORD,USER	breed	2.129731	WORD
9	verification	WORD,USER	changer	2.129731	WORD
9	verification	WORD,USER	code-to-model	2.129731	WORD
9	verification	WORD,USER	coupon	2.129731	WORD
9	verification	WORD,USER	demo	2.129731	WORD
9	verification	WORD,USER	emphasise	2.129731	WORD
9	verification	WORD,USER	expedition	2.129731	WORD
9	verification	WORD,USER	hypothetico-deductive	2.129731	WORD
9	verification	WORD,USER	lenders	2.129731	WORD
9	verification	WORD,USER	listen	2.129731	WORD
9	verification	WORD,USER	nights	2.129731	WORD
9	verification	WORD,USER	opt	2.129731	WORD
9	verification	WORD,USER	pink	2.129731	WORD
9	verification	WORD,USER	refugee	2.129731	WORD
9	verification	WORD,USER	source-code	2.129731	WORD
